# Treatment-Emergent Bictegravir Resistance in a Treatment-Naive Individual With HIV Infection: A Case Report

**DOI:** 10.7759/cureus.106218

**Published:** 2026-03-31

**Authors:** Iya Dubson, Aahana Gaur, Jasmin Jose, Charles Andrews, Andrew Gaballa, Samy I McFarlane

**Affiliations:** 1 Infectious Disease, Department of Veterans Affairs Medical Center, New York Harbor Healthcare System, Brooklyn Campus, Brooklyn, USA; 2 Pharmacy, Department of Veterans Affairs Medical Center, New York Harbor Healthcare System, Brooklyn Campus, Brooklyn, USA; 3 Medicine, State University of New York Downstate Health Sciences University, Brooklyn, USA

**Keywords:** bictegravir, hiv disease, insti resistance, treatment naive, treatment resistance

## Abstract

Bictegravir/emtricitabine/tenofovir alafenamide (BIC/FTC/TAF) is a highly potent antiretroviral regimen with a high genetic barrier to resistance. Clinical trials have not reported treatment-emergent resistance in treatment-naive individuals. We describe a rare case of virologic failure with an emergent R263K integrase mutation in a treatment-naïve, adherent individual maintained on BIC/FTC/TAF. The patient achieved initial suppression for nearly two years before rebound viremia with R263K and D67N mutations. Following the regimen change and treatment of concurrent syphilis, viral suppression was re-established. This case highlights that emergent bictegravir resistance can occur in adherent, treatment-naïve patients, emphasizing the importance of continued resistance monitoring.

## Introduction

Human immunodeficiency virus (HIV) remains a major global health challenge, with 40 million people living with the virus, 1.3 million new infections, and 630,000 deaths in 2024 [1]. Though antiretroviral therapy (ART) has transformed HIV into a manageable condition, only 73% of those infected achieve viral suppression [1]. Sustained suppression is critical to preserving immune function, preventing AIDS, and eliminating transmission risk. Virologic failure (HIV RNA ≥ 200 copies/mL on ART) requires prompt evaluation for drug resistance [2].

Drug resistance mutations (DRMs) arise under subtherapeutic drug levels or sustained viral replication, with impact depending on mutation class, cross-resistance, and viral fitness cost. Nucleoside reverse transcriptase inhibitor (NRTI), non-nucleoside reverse transcriptase inhibitor (NNRTI), and protease inhibitor (PI) resistance were historically common with older, lower-barrier regimens. Second-generation integrase strand transfer inhibitors (INSTIs), bictegravir and dolutegravir, markedly reduced treatment-emergent resistance through their high genetic barrier, requiring multiple simultaneous mutations for meaningful loss of susceptibility [3].

Bictegravir/emtricitabine/tenofovir alafenamide (BIC/FTC/TAF) is a fixed-dose combination regimen approved for adults and pediatric patients with HIV-1 infection who are treatment-naive or virologically suppressed [2]. Bictegravir is a second-generation INSTI characterized by potent antiviral efficacy and a high genetic barrier to resistance [2,4]. Emtricitabine is a cytidine analogue NRTI that inhibits HIV reverse transcriptase and causes chain termination. Tenofovir alafenamide is a tenofovir prodrug that achieves high intracellular concentrations at lower plasma levels than tenofovir disoproxil fumarate, improving renal and bone safety while maintaining antiviral efficacy [5]. In clinical trials and real-world studies, BIC/FTC/TAF showed noninferiority to comparators in treatment-naive patients, with no emergent resistance observed [6,7].

Resistance to first-generation INSTIs such as raltegravir and elvitegravir typically arises through Q148, N155, or G140 pathways, whereas the R263K substitution, observed with dolutegravir and bictegravir, confers low-level resistance accompanied by a significant viral fitness cost, which is thought to limit its clinical prevalence despite ongoing selective pressure [8-11]. Additionally, D67N, a thymidine analogue mutation (TAM) historically associated with zidovudine and stavudine, has also been identified in this case and remains classified in current resistance guidelines [12].

We report a case of treatment-emergent bictegravir resistance in a treatment-naive patient with excellent adherence.

## Case presentation

A 30-year-old cisgender male with newly diagnosed HIV presented in September 2021 after unprotected sexual contact. Baseline HIV-1 RNA was 1,793,232 copies/mL; CD4 count was 335 cells/mm³. The baseline genotype revealed no NRTI, NNRTI, or PI resistance mutations; integrase testing was not performed at baseline.

He was started on BIC/FTC/TAF, and he showed excellent adherence. The viral load became undetectable by September 2022 and remained suppressed through July 2024, except for a one-month interruption in July 2023 due to refill delays. The viral load was re-suppressed promptly after therapy was resumed.

In July 2024, he presented with constitutional symptoms, lymphadenopathy, and bilateral hearing loss. Laboratory evaluation revealed virologic rebound with an HIV RNA of 163,718 copies/mL and a CD4 count of 328 cells/mm³. Given his previously sustained viral suppression and documented adherence, treatment-emergent resistance was suspected, prompting genotypic resistance testing. A concurrent workup revealed a reactive RPR at 1:128 and a positive rectal gonorrhea swab. The combination of high-titer RPR and bilateral hearing loss prompted evaluation and treatment for otosyphilis.

Genotypic resistance testing (GenoSure Prime), as shown in Table 1, identified D67D/N in the NRTI class, E35D (a polymorphic substitution) in the protease gene, and R263R/K in the integrase inhibitor class. The detection of mixed viral populations at both the NRTI (D67D/N) and INSTI (R263R/K) loci is more consistent with in vivo emergence of resistance under ongoing selective drug pressure than acquisition through superinfection, though the latter cannot be entirely excluded. The R263K mutation confers reduced susceptibility (~2-fold) to bictegravir, dolutegravir, and cabotegravir, while its presence antagonizes the development of raltegravir resistance, potentially preserving raltegravir as a salvage option in the absence of additional integrase mutations [12].

**Table 1:** Genotypic resistance testing and its clinical significance. Abbreviations: NRTI, nucleoside reverse transcriptase inhibitor; PI, protease inhibitor; INSTI, integrase strand transfer inhibitor; TAM, thymidine analogue mutation. Source: [12].

Mutation	Drug Class	Predicted Susceptibility	Clinical Significance
D67D/N	NRTI	Reduced susceptibility to most NRTIs; greater resistance when combined with other TAMs	Classic thymidine analogue mutation (TAM), historically selected by zidovudine and stavudine, confers decreased susceptibility to most NRTIs through enhanced ATP-mediated excision of chain terminators. Detection of a mixed D67D/N population is consistent with in vivo emergence of resistance under ongoing selective drug pressure.
E35D	PI	Susceptible	Polymorphic hinge-region substitution in the protease gene; not associated with protease inhibitor resistance. Naturally occurring variant with no clinical impact on treatment.
R263K	INSTI	Low-level reduced susceptibility (~2-fold) to bictegravir, dolutegravir, and cabotegravir; minimal impact on raltegravir and elvitegravir.	Non-polymorphic INSTI-selected mutation; impairs integrase-DNA binding and reduces viral fitness. R263K antagonizes raltegravir resistance development, potentially preserving raltegravir as a salvage option in the absence of additional integrase mutations. Detection of a mixed R263R/K population is consistent with in vivo emergence of resistance under selective drug pressure.

Given the identified resistance profile, his regimen was switched from BIC/FTC/TAF to emtricitabine/rilpivirine/tenofovir alafenamide (FTC/RPV/TAF), substituting the INSTI component with rilpivirine, a second-generation NNRTI. Full viral suppression was achieved at one month. He completed ceftriaxone 2 g IV daily for 14 days for otosyphilis, with full resolution of hearing loss and systemic symptoms.

## Discussion

Emergent resistance to bictegravir is exceedingly rare. Clinical trials and cohort studies have consistently shown a high resistance barrier with no INSTI resistance among treatment-naive individuals [6,7,13]. Real-world data have further confirmed this efficacy profile, including in populations with suboptimal adherence and pre-existing resistance mutations [13], highlighting that the circumstances leading to resistance in our patient are distinctly unusual.

The R263K mutation is well described in vitro and in association with dolutegravir failure, often under selective pressure from poor adherence or drug interactions [8-10]. Quashie et al. first characterized R263K as the predominant mutation selected by dolutegravir in in vitro selection experiments across multiple HIV-1 subtypes, demonstrating that it reduces integrase-DNA binding affinity and strand transfer activity, thereby decreasing both viral replicative capacity and integrase enzymatic efficiency [11]. This fitness cost is thought to limit the clinical prevalence of R263K: despite its selection under second-generation INSTI pressure, it confers only a low-level (~2-fold) reduced susceptibility to bictegravir and dolutegravir, and the combination of R263K with M184I/V further compounds the replicative fitness deficit [11].

Although not observed in treatment-naïve clinical trials, a few case reports have described bictegravir resistance in clinical practice. Lozano et al. reported a treatment-naive patient who developed the R263K mutation following virologic failure on BIC/FTC/TAF; however, resistance emergence in that case was attributed to poor adherence, with documented treatment interruptions preceding failure [14]. Furthermore, Stoll et al. reported emergent INSTI resistance during first-line bictegravir therapy, noting preserved viral replication and possible contributing factors, including severe immunodeficiency, high baseline viral load, and non-adherence [15]. In a different clinical context, Chamberlain et al. described emergent bictegravir resistance in a heavily treatment-experienced patient receiving bictegravir as part of salvage therapy, where prior antiretroviral exposure and cumulative resistance likely contributed to resistance development [16]. In contrast, our patient had no prior ART exposure, sustained suppression for nearly two years, and self-reported excellent adherence.

The emergence of R263K in a treatment-naive patient with documented adherence is unusual and raises the theoretical possibility of superinfection with a resistant strain, as described in the literature [17]. However, our patient’s long period of viral suppression and the detection of mixed viral populations at both resistance loci (D67D/N and R263R/K) argue for in vivo selection rather than replacement of the infecting strain through superinfection with a fully resistant virus. Mixed populations on standard genotyping indicate that both wild-type and mutant variants are circulating simultaneously. Genotypic testing has greater sensitivity for detecting mixtures of wild-type and resistant virus compared to phenotypic testing, and drug-resistant viruses constituting <10-20% of the circulating population may not be detected by commercially available assays [2].

The concurrent emergence of D67N alongside R263K is also noteworthy. The D67N mutation is a classic TAM historically selected by zidovudine and stavudine. TAMs confer decreased susceptibility to most NRTIs through enhanced ATP-mediated excision of chain terminators from the 3' end of the terminated DNA strand [18]. Although D67N alone confers only modest resistance, its clinical significance increases when combined with other TAMs (M41L, K70R, L210W, T215Y/F, K219Q/E). Notably, TAMs can also promote cross-resistance to tenofovir, particularly when three or more TAMs are present, including M41L or L210W [19]. The detection of a mixed D67D/N population in our patient, who was never exposed to thymidine analogues, suggests either de novo selection under tenofovir pressure or, less likely, transmitted resistance that remained archived.

## Conclusions

We present one of the few documented cases of treatment-emergent bictegravir resistance in a treatment-naive, adherent patient. While rare, R263K can emerge even with good adherence, showing that high-barrier regimens do not preclude resistance in all clinical scenarios. Routine viral load monitoring, prompt resistance testing at virologic failure, and individualized regimen selection remain essential even with high-barrier contemporary antiretroviral therapy.
